# Multidimensional cfRNA response modeling identifies a 5-gene pair signature for high-robust pulmonary tuberculosis diagnosis

**DOI:** 10.1016/j.isci.2026.115178

**Published:** 2026-02-28

**Authors:** Changchun Wu, Huiyuan Qiao, Xianrun Pan, Hongyan Lin, Xueqin Xie, Mengze Du, Hao Lin, Jian Huang

**Affiliations:** 1 The Clinical Hospital of Chengdu Brain Science Institute, School of Life Science and Technology, University of Electronic Science and Technology of China, Chengdu 611731, China; 2 School of Healthcare Technology, Chengdu Neusoft University, Chengdu 611844, China

**Keywords:** Health sciences

## Abstract

Lack of non-invasive biomarkers hinders pulmonary tuberculosis (PTB) management. We developed a multidimensional machine learning framework to systematically evaluate five cell-free RNA (cfRNA)-derived host response modalities: immune cell infiltration, global transcriptional perturbation (expression- and rank-based), key genes, and gene pairs. While immune-cell and global perturbation models showed moderate efficacy, an optimized 5-gene pair classifier demonstrated robust diagnostic power. This signature achieved an AUC of 0.947 in the validation subset derived from the parent study and maintained 100% sensitivity (95% CI: 0.741–1.000) in HIV-coinfected individuals. Model scores correlated significantly with bacterial load (*r* = 0.65) and radiological severity. Kappa analysis confirmed substantial diagnostic agreement (kappa = 0.42–0.56) between this signature and chest X-ray systems, with the signature providing high discriminative power in cases with ambiguous radiological findings. This cfRNA-based 5-gene pair signature establishes a robust diagnostic paradigm, offering efficient PTB detection in resource-limited settings and a foundation for future point-of-care diagnostics.

## Highlights

• Multinational cfRNA profiling reveals gender-independent PTB signatures
• Patients with PTB exhibit innate immunity with neutrophil and mast cell activation
• A 5-gene pair Naive Bayes classifier achieves high accuracy (AUC = 0.947)
• Scores correlate with bacterial load and X-ray severity, supporting clinical utility

## Graphical abstract

**Figure:**
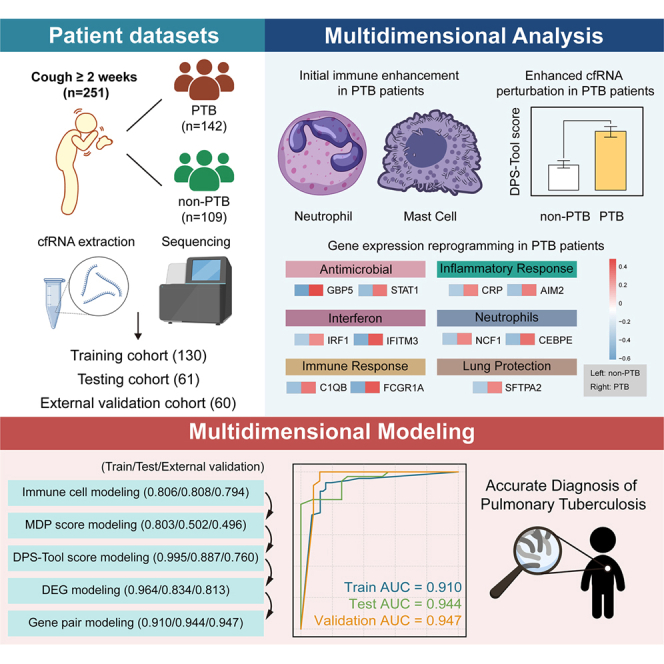

## Introduction

Pulmonary tuberculosis (PTB), caused by *Mycobacterium tuberculosis* (MTB), remains a leading global cause of infectious disease mortality.^1^^,^^2^^,^^3^ According to the Global Tuberculosis Report 2024,^4^^,^^5^ approximately 10.8 million new tuberculosis cases and 1.25 million deaths were reported worldwide in 2023, posing a serious threat to global public health. Early diagnosis remains critical for effective PTB control in high-burden countries. However, current diagnostic techniques face substantial limitations: Sputum smear microscopy exhibits low sensitivity,^6^ while molecular assays such as Xpert MTB/RIF show restricted applicability for patients with inadequate sputum samples.^7^^,^^8^ This highlights the urgent need for novel, sputum-independent biomarkers that can identify PTB through systemic signals.

Transcriptomic profiling, as a molecular fingerprint of host immune responses, has been introduced as a promising diagnostic target.^9^^,^^10^ Although whole-blood RNA (wbRNA) biomarkers have been extensively explored,^11^^,^^12^^,^^13^^,^^14^ their predominant derivation from circulating immune cells constrains their ability to fully reflect localized or tissue-specific host responses (e.g., signals from organ injury), which may contribute to the difficulty in meeting the World Health Organization (WHO)-recommended performance targets for diagnostic accuracy.^6^^,^^15^ In recent years, plasma cell-free RNA (cfRNA) has emerged as a complementary approach that contains information about systemic immune dynamics and immune-tissue interactions.^16^ For instance, in a large multinational PTB cohort, Chang et al.^17^ demonstrated that a 6-gene cfRNA panel achieved robust performance in distinguishing PTB-positive from PTB-negative individuals, comparing favorably to existing wbRNA panels. Nonetheless, the 6-gene cfRNA panel showed limited specificity in the validation cohort (76.0%), and its absolute expression values were susceptible to batch effects introduced by technical variability, including RNA extraction and reverse transcription bias, as well as sequencing platform heterogeneity, which may impair reproducibility across centers.

In this study, we developed a machine learning framework integrating gene expression rank-based relationships to identify cfRNA-derived gene pair biomarkers for PTB diagnosis, building upon a systematic re-analysis of established clinical cfRNA datasets. By comparatively evaluating multiple diagnostic modalities, including immune cell infiltration, transcriptome perturbation, absolute expression abundance, and gene-pair features, we demonstrated that our gene-pair-based strategy significantly optimizes diagnostic accuracy. In the parent-study validation cohort, the identified gene-pair model achieved a specificity of 84.0% (an 8.0% improvement over the previously established 6-gene signature) with an overall AUC of 0.947, highlighting its enhanced technical stability across diverse datasets. Importantly, gene-pair features, based on relative gene expression ordering, exhibited exceptional technical robustness, thereby providing a robust methodological foundation for the application of cfRNA-based PTB diagnosis.

## Results

### Host immune cell infiltration and sex-independent cell-free RNA profiles in pulmonary tuberculosis

**Figure 1:**
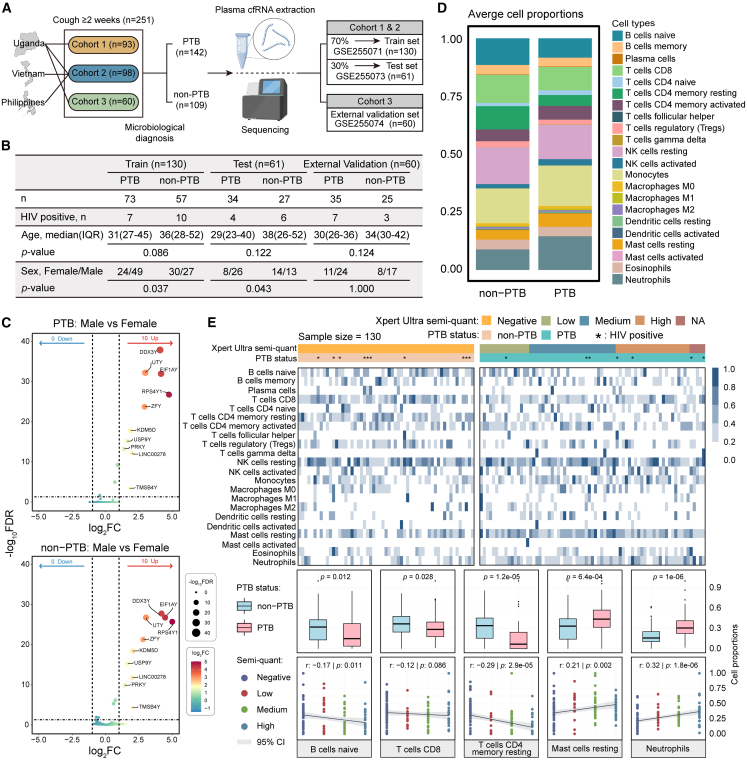
Overall characterization of the PTB cohort and immune infiltration landscape (A) Sources and distribution of cohort data across the training, test, and validation sets. (B) Summary statistics of key clinical characteristics in the three datasets, with age and sex differences evaluated using Wilcoxon rank-sum and chi-square tests, respectively. (C) Sex-associated differential expression analysis: differentially expressed genes (DEGs) between males and females in PTB samples (top) and in non-PTB samples (bottom), defined as adjusted *p*-value <0.05 and |log2 fold change| > 1; dot size and color represent adjusted *p*-value and log2 fold change, respectively. (D) Stacked bar plot shows average proportions of 22 immune cell types in PTB versus non-PTB groups. (E) Immune infiltration analysis of the 22 cell types: heatmap of relative abundance across PTB and non-PTB samples (top), Wilcoxon rank-sum test results highlighting significant differences (middle, PTB *n* = 73, non-PTB *n* = 57), and Kendall rank correlation analysis of five cell types with Xpert Ultra semi-quantitative grades (bottom; gray shadows represent 95% confidence intervals).

A multi-country cohort including participants from Uganda, Vietnam, and the Philippines was stratified into training (*n* = 130), testing (*n* = 61), and validation sets (*n* = 60) according to the original study protocol (Figure 1A and Table S1).^17^ Statistical analyses revealed comparable age distributions between PTB and non-PTB groups across all datasets (Figure 1B). While significant male predominance was observed in PTB groups (Figure 1B: *p* < 0.05), consistent with global epidemiological trends,^4^^,^^18^^,^^19^ differential cfRNA analysis revealed that PTB host responses are largely gender-independent. Only 10 Y-chromosome-linked genes (e.g., *RPS4Y1* and *EIF1AY*) exhibited sex-specific expression, with no discriminative molecular signals detected from autosomes (Figure 1C). These findings suggest that sex disparities in PTB are more likely linked to extrinsic factors rather than intrinsic molecular mechanisms, indicating that cfRNA-based diagnostics may not require sex-specific adaptation.

We further characterized the immune cell infiltration landscape between the PTB and non-PTB groups. PTB samples exhibited reduced levels of naive B cells, CD8^+^ T cells, and resting memory CD4 T+ cells, alongside elevated neutrophils and resting mast cells, indicating enhanced innate immune activation in patients with PTB (Figures 1D and 1E).^20^^,^^21^^,^^22^ These shifts were closely associated with pathogen burden, as naive B cell and resting memory CD4^+^ T cell abundances declined with increasing Xpert Ultra grades,^23^^,^^24^ while neutrophils and mast cells increased (Figure 1E). Additionally, elevated NK cells, Monocytes, and M1 macrophages were observed (Figure 1D), characterizing a complex immune microenvironment and dynamic immune remodeling associated with PTB.

### Pulmonary tuberculosis cell-free RNA exhibits a significant molecular degree of perturbation

**Figure 2:**
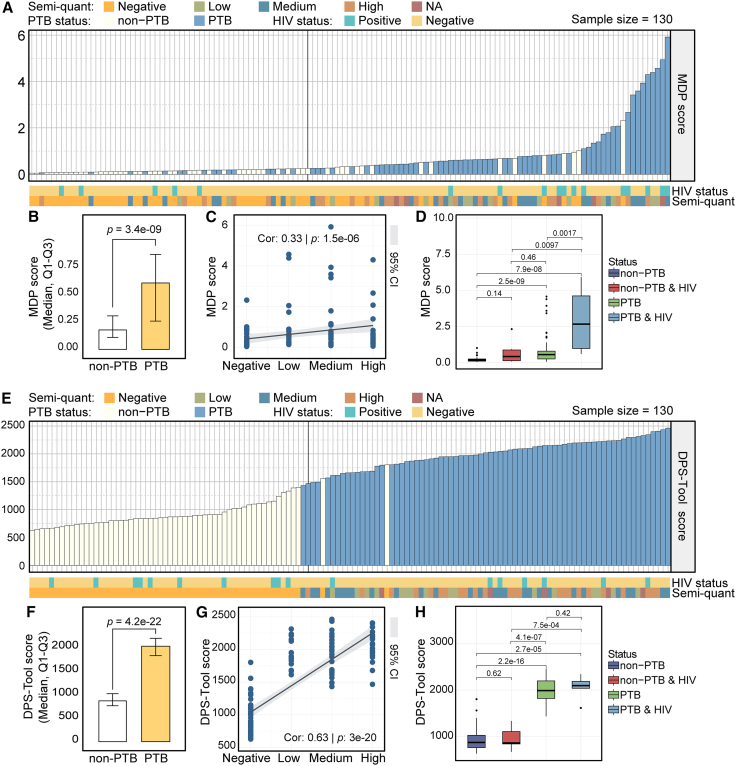
Analysis of molecular degree of perturbation in PTB (A) Training set samples (*n* = 130) ranked in ascending order according to expression value-based perturbation scores. The black vertical dashed line at the 57th sample marks the expected boundary between the 57 non-PTB and 73 PTB samples based on the cohort composition. This reference line facilitates a visual evaluation of classification accuracy, where samples appearing on the incorrect side of the boundary represent misclassifications (e.g., PTB samples appearing before the line or non-PTB samples appearing after it). (B) Bar plot compares expression value-based perturbation scores between PTB (*n* = 73) and non-PTB (*n* = 57) samples. Bar height represents the median perturbation score. Two-sided Wilcoxon rank-sum test. (C) Correlation between expression value-based perturbation scores and semi-quantitative Xpert Ultra results. Kendall rank correlation test. The gray shadows represent the 95% confidence interval. (D) Comparison of expression value-based perturbation scores across four groups stratified by PTB and HIV co-infection status: non-PTB (*n* = 47), non-PTB & HIV (*n* = 10), PTB (*n* = 66), and PTB & HIV (*n* = 7). Two-sided Wilcoxon rank-sum test. (E) Training set samples (*n* = 130) ranked in ascending order according to rank-based perturbation scores. The black vertical dashed line at the 57th sample marks the expected boundary between the 57 non-PTB and 73 PTB samples based on the cohort composition. (F) Bar plot comparing rank-based perturbation scores between PTB (*n* = 73) and non-PTB (*n* = 57) samples. Bar height indicates the median score. Two-sided Wilcoxon rank-sum test. (G) Correlation between rank-based perturbation scores and semi-quantitative Xpert Ultra results. Kendall rank correlation test. The gray shadows represent the 95% confidence interval. (H) Comparison of rank scores among four groups stratified by PTB and HIV status: non-PTB (*n* = 47), non-PTB & HIV (*n* = 10), PTB (*n* = 66), and PTB & HIV (*n* = 7). Two-sided Wilcoxon rank-sum test.

To assess the systemic impact of PTB on plasma cfRNA, we calculated the global transcriptional perturbation features for each sample.^25^^,^^26^^,^^27^ Perturbation scores were markedly elevated in PTB compared with non-PTB individuals (Figures 2A and 2B), indicating a widespread disruption of peripheral cfRNA expression profiles associated with PTB. Furthermore, the perturbation scores progressively increased with bacterial load (Figure 2C: Xpert Ultra semiquantitative grades; *r* = 0.33, *p* = 1.5e-6), indicating that transcriptional perturbation is closely associated with pathogen burden. Next, we divided the samples into four groups based on PTB and human immunodeficiency virus (HIV) infection status: non-PTB, non-PTB & HIV, PTB, and PTB & HIV (Figure 2D). The PTB & HIV coinfection individuals had the highest perturbation scores, while non-infected controls showed the lowest scores. Notably, molecular perturbation analysis using expression values could not distinguish HIV-positive non-PTB individuals from patients with HIV-negative PTB (Figure 2D, *p* = 0.46), indicating that these scores may be insufficient to differentiate PTB-specific pathological signals from HIV-associated immune activation.

To address this and obtain a PTB-specific transcriptional perturbation profile, we applied molecular perturbation analysis based on expression rankings using a gene pair feature strategy (see STAR Methods). In the training set of 130 samples, sorting perturbation scores obtained with expression values and stratifying by PTB status resulted in 16 of 57 non-PTB samples being misclassified as PTB and 16 of 73 PTB samples being misclassified as non-PTB (Figure 2A). In contrast, the error rates for rank-based perturbation scores were only 2/57 and 2/73, respectively (Figure 2E), demonstrating that rank-based global perturbation features provide a more accurate and robust assessment compared with expression-based features. The rank-based perturbation scores exhibited stronger intergroup discrimination (*p* = 4.2e-22), lower intragroup variance (Figure 2F), and a higher correlation with bacterial load (Figure 2G: *r* = 0.63, *p* = 3e-20). Importantly, rank-based scores successfully distinguished HIV-positive non-PTB individuals from patients with HIV-negative PTB (Figure 2H), demonstrating their ability to mitigate HIV-related confounding and selectively capture PTB-specific transcriptional signatures.

### Upregulation of inflammation and innate immunity-associated cell-free RNA in pulmonary tuberculosis samples

**Figure 3:**
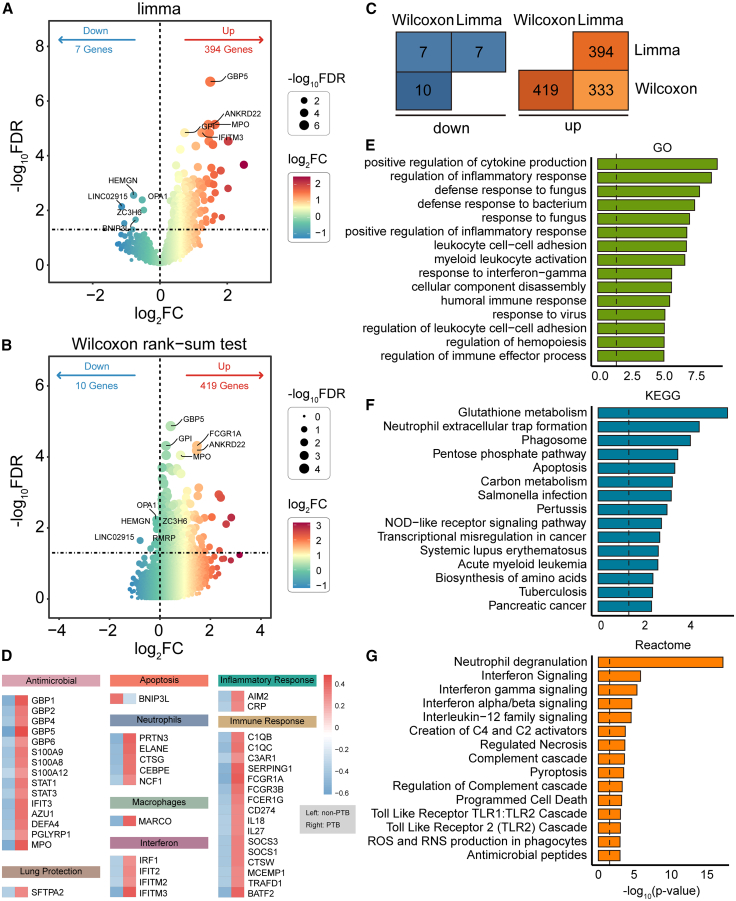
Differential expression analysis of cfRNA in PTB samples (A and B) Volcano plots show differentially expressed cfRNAs between PTB and non-PTB samples. Differential genes were defined by FDR <0.05. Dot size and color represent FDR and log_2_ fold change, respectively. Differential expression was identified using limma (A) and the Wilcoxon rank-sum test (B). (C) Overlap statistics of DEGs identified by the two methods. Orange indicates upregulated genes, and blue represents downregulated genes. (D) Expression patterns of key module-related genes among the 340 overlapping DEGs. The color scale represents the mean expression level within each sample group. (E and G) Functional enrichment analysis of the 340 overlapping DEGs. The black dashed line indicates a significance threshold of *p* < 0.05.

To further characterize the specific features of cfRNA alterations in patients with PTB, we performed differential expression analysis using limma and the Wilcoxon rank-sum test to compare cfRNA profiles between PTB and non-PTB individuals. Limma identified 394 upregulated and 7 downregulated genes in PTB (FDR <0.05; Figure 3A and Table S2), including key innate immunity and inflammation-related genes (e.g., *GBP5*) and interferon-related genes such as *IFITM3*. Wilcoxon analysis yielded highly consistent results (FDR <0.05: 419 upregulated, 10 downregulated; Figures 3B and Table S3). A substantial overlap of 333 upregulated and 7 downregulated genes was observed between the two methods (Figure 3C), indicating robust transcriptional changes in PTB. Functional categorization of the 340 overlapped genes revealed significant upregulation of antimicrobial (*GBP* family), interferon signaling (*IRF* family), and lung-specific markers (*SFTPA2*), reflecting cfRNA complexity and infection-driven host remodeling (Figure 3D and Table S4).

To gain insight into the functional relevance of these cfRNA alterations, we conducted enrichment analysis on the 340 overlapping genes. The results showed significant enrichment in pathways related to inflammatory response, cytokine regulation, interferon signaling, myeloid immune activation, phagosome formation, and apoptosis (Figures 3E–3G). Kyoto Encyclopedia of Genes and Genomes (KEGG) and Reactome analyses further emphasized neutrophil extracellular traps (NETs), NOD-like receptor signaling, Toll-like receptor cascades, and complement activation, underscoring innate immunity as a central feature in PTB cfRNA. Collectively, these results demonstrated a convergent upregulation of innate immune and inflammatory signals in PTB cfRNA, supporting its potential as a biomarker for active tuberculosis infection.

### Comparative analysis of multimodal diagnostic models reveals the superiority of gene pair features

To identify the most robust biomarkers for PTB diagnosis, we systematically compared diagnostic models across five feature modalities: immune cell infiltration, expression value-based transcriptional perturbation scores, rank-based perturbation scores, key differentially expressed genes (DEGs), and gene pair features.

**Figure 4:**
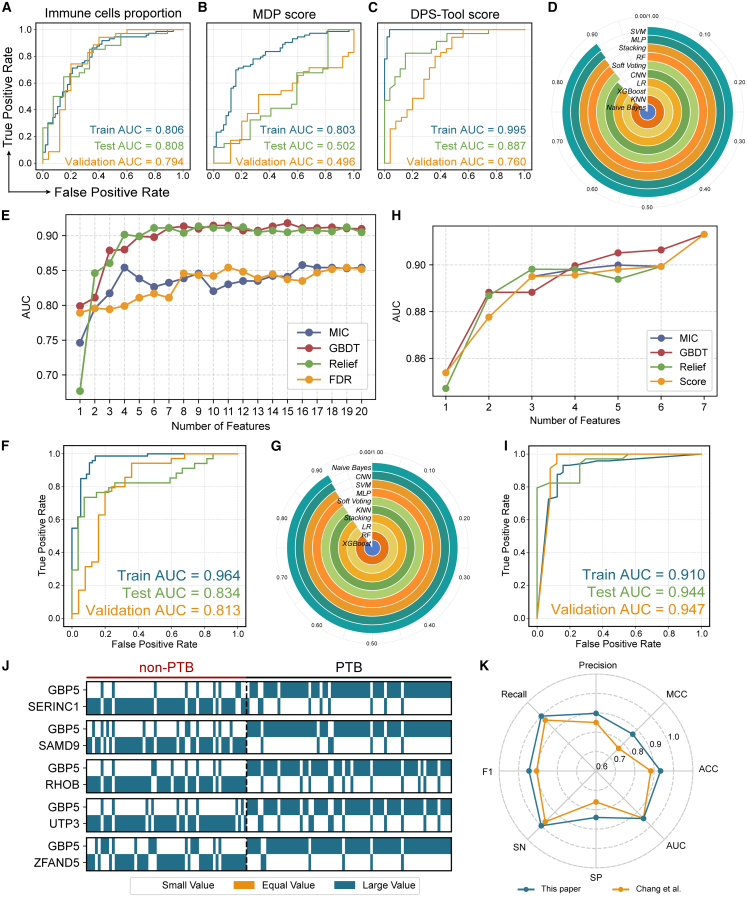
Construction of PTB diagnostic models based on different feature modalities (A–C) PTB diagnostic models built using LR with default parameters, based on immune cell infiltration features (A), global transcriptional perturbation scores based on expression values (B), and rank-based global perturbation scores (C). (D) Performance comparison of models constructed using 45 key DEGs as input features across 10 classification algorithms with 5-fold cross-validation (CV) on the training set. All models used default parameters. (E) IFS process of the SVM-based model using the 45 DEGs. Only the top 20 feature inclusion steps are shown. The AUC of 5-fold CV on the training set was used for performance evaluation. The model reached a performance inflection point when the top 4 DEGs selected by the Relief method were included. (F) Prediction performance of the model based on the top 4 DEGs with SVM. (G) Comparison of model performance using 7 gene pair features across 10 classification algorithms with 5-fold CV on the training set, all using default parameters. (H) IFS process for the Naive Bayes-based model using the 7 gene pair features. Performance evaluated via 5-fold cross-validation. The top 5 gene pairs selected by the GBDT method marked the inflection point. (I) Prediction performance of the model constructed using the top 5 gene pairs and Naive Bayes. (J) Expression ranking patterns of the top 5 gene pairs (selected by GBDT) in PTB and non-PTB samples. (K) Radar plot comparing the performance of the 5 gene pair-based model from this study and the 6-gene model proposed by Chang et al. across 8 evaluation metrics on the validation cohort. Note: Performance metrics for the 6-gene model, including AUC, ACC, SN/Recall, and SP, were obtained directly from the values reported in the original study by Chang et al. Additional metrics not explicitly provided in the original text, including the Matthews Correlation Coefficient (MCC), F1 score, and Precision, were calculated based on the known sample distribution (35 positive and 25 negative cases) and the associated performance metrics (sensitivity and specificity).

We first evaluated global biological profiles, including immune cell and global transcriptional perturbation. We used five immune cell types that showed significant abundance differences between PTB and non-PTB groups, including naive B cells, CD8^+^ T cells, CD4^+^ memory resting T cells, resting mast cells, and neutrophils, as input features to train a logistic regression (LR)-based predictive model. The model yielded moderate performance (Figure 4A: AUCs: 0.794–0.808), indicating that while immune infiltration reflects PTB-related responses, its limited predictive power precludes high-precision diagnosis in complex clinical settings. To evaluate global transcriptome shifts, we compared absolute expression-based versus rank-based perturbation scores. The perturbation scores for the testing and validation cohorts were calculated using the perturbation parameters learned from the training cohort to ensure consistency. The absolute expression LR-based model failed significantly upon cross-dataset validation (Figure 4B: AUC_training_ = 0.803, AUC_testing_ = 0.502, AUC_validation_ = 0.496), suggeting severe batch effects and overfitting. In contrast, the rank-based model, which captures gene order disruptions, mitigated batch effects and achieved a validation AUC of 0.760 (Figure 4C).

To address the dimensionality and noise susceptibility of global models, we then focused on targeted local features. For the DEG-based modality, we initially screened 45 key candidates (Figure 3D) and tested ten classification algorithms, among which the support vector machine (SVM)-based model demonstrated the highest initial discriminative power (Figure 4D). To further optimize this model, we applied Incremental Feature Selection (IFS) combined with four feature importance methods (Table S5): maximal information coefficient (MIC), gradient boosting decision tree (GBDT), Relief, and false discovery rate (FDR). By identifying the performance inflection point, we reduced the feature set to the top 4 genes (relief-based method), resulting in a finalized SVM-based model with AUCs of 0.964, 0.834, and 0.813 in training, testing, and validation sets (Figures 4E and 4F).

Considering the susceptibility of absolute expression values to technical noise, we further explored whether local transcriptome features based on a gene-pair framework could enhance PTB diagnostic performance. To determine the optimal feature selection criteria, we evaluated a range of disparity score thresholds. As shown in Table S6, decreasing the threshold significantly increased the number of candidate gene pairs (e.g., from 1 pair at 0.70 to 2,317 pairs at 0.50), which could lead to model overfitting and reduced clinical interpretability. Ultimately, a threshold of 0.65 was selected to balance biological relevance and feature parsimony, yielding 7 high-confidence gene pairs for subsequent diagnostic modeling (Table S7). To identify the most robust diagnostic markers, we conducted a systematic model and feature selection process, following the same pipeline as the local DEG-based model. Among ten classification models evaluated using the candidate gene pairs, the Naive Bayes algorithm demonstrated the superior predictive performance (Figure 4G). Then, we ranked the gene pairs using four independent feature importance methods (Table S8). By employing IFS, we observed that the model’s discriminative power reached an inflection point with the inclusion of the top 5 gene pairs as ranked by the GBDT method (Figure 4H). The final diagnostic model was constructed using the top 5 specific gene pairs: *GBP5-SERINC1, GBP5-SAMD9, GBP5-RHOB, GBP5-ZFAND5*, and *GBP5*-*UTP3* (Figure 4J). These pairs involve 6 unique genes primarily associated with interferon-inducible innate immune signaling and intracellular vesicle trafficking (Table S9). This 5-gene pair model demonstrated robust cross-cohort discriminative power between PTB and non-PTB, achieving AUCs of 0.910 (training), 0.944 (testing), and 0.947 (validation) (Figure 4I). These results suggest the high potential of local gene-pair features for PTB diagnosis compared to the other modalities evaluated in this study.

### Diagnostic model with a 5-gene pair signature significantly enhances specificity in pulmonary tuberculosis detection

**Figure 5:**
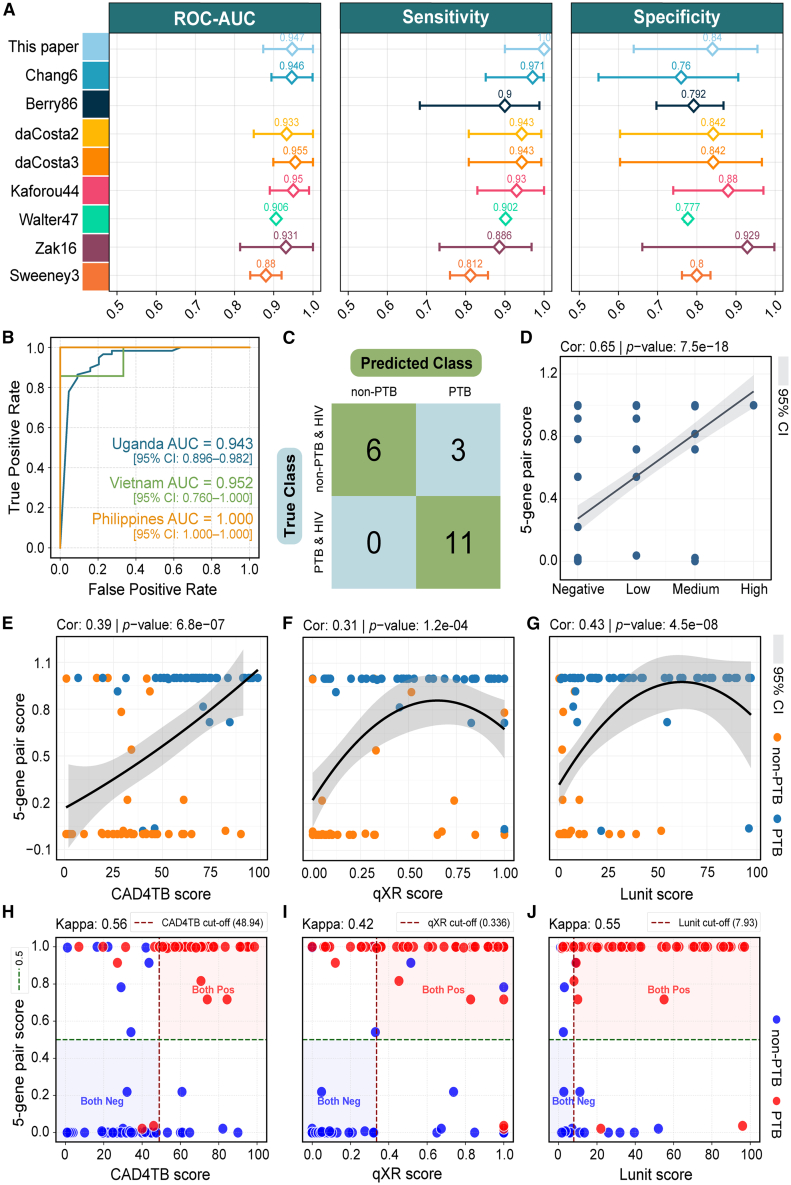
Evaluation and validation of the 5-gene pair diagnostic model (A) Performance comparison of whole blood signatures from previous multi-cohort studies and Chang’s 6-gene cfRNA with our 5-gene pair cfRNA signature. Error bars represent the 95% confidence intervals. (B) Model performance across three countries after merging the testing and validation cohorts. The samples were stratified by country: Uganda (PTB: *n* = 59, non-PTB: *n* = 44), Vietnam (PTB: *n* = 7, non-PTB: *n* = 3), and the Philippines (PTB: *n* = 3, non-PTB: *n* = 5). (C) Confusion matrix evaluates the model’s performance in identifying PTB cases among HIV co-infected individuals, based on the combined testing and validation datasets (HIV-positive PTB: *n* = 11, HIV-positive non-PTB: *n* = 9). Note: For the HIV-coinfected subgroup, the 95% CIs are explicitly reported (Sensitivity: 100% [95% CI: 0.741–1.000]; Specificity: 66.7% [95% CI: 0.354–0.879]) to reflect the statistical uncertainty associated with the small sample size. (D) Correlation between the model’s predicted probability scores and Xpert Ultra semi-quantitative results. The analysis was performed on the merged testing and validation datasets using the Kendall rank correlation test. Gray shading indicates the 95% confidence interval. (E–G) Correlation between the model’s predicted probability scores and three chest X-ray scoring systems (CAD4TB, qXR, and Lunit). Colors represent disease status. Analyses were conducted using the Kendall rank correlation test on the merged testing and validation datasets (PTB: *n* = 47, non-PTB: *n* = 40), with gray shading indicating the 95% confidence interval. (H–J) Diagnostic consistency and Cohen’s Kappa analysis. To evaluate the model in a binary (“yes/no”) diagnostic framework, study-specific optimal cut-offs for chest X-ray AI systems were determined based on the maximum Youden Index within the cohort, yielding thresholds of 48.94 for CAD4TB, 0.336 for qXR, and 7.93 for Lunit. These radiological thresholds are compared against the fixed 5-gene pair model threshold of 0.5 (indicated by green dashed lines), with the four quadrants representing diagnostic agreement (Both Pos and Both Neg) and discordant zones.

To further validate diagnostic superiority, we systematically compared our 5-gene pair model with Chang et al.’s 6-gene model^17^ in the validation cohort. Across eight evaluation metrics (Figure 4K and Table S10), our model outperformed the comparator in all metrics, demonstrating a robust and comprehensive diagnostic advantage. The most prominent improvements were observed in specificity and Matthews Correlation Coefficient (MCC): specificity increased from 0.760 to 0.840, and MCC rose from 0.765 to 0.868, indicating substantial improvements in reducing misdiagnosis and enhancing classification accuracy. Beyond comparison within the cfRNA modality, we further evaluated the added value of our cfRNA-based model relative to established whole blood (wb) RNA diagnostic signatures. While numerous wbRNA signatures have been developed for TB diagnosis, their performance often exhibits significant heterogeneity across diverse populations. Among the wbRNA signatures, we selected seven high-performance panels, including Berry86,^28^ daCosta2,^29^ daCosta3,^29^ Kaforou44,^30^ Walter47,^31^ Zak16,^13^ and Sweeney3,^11^ for a comprehensive comparison. As shown in Figure 5A, our 5-gene pair cfRNA model compares favorably against these established wbRNA signatures across multiple performance metrics.

We next assessed the generalizability of the model across different countries. By merging the testing and validation cohorts and stratifying samples by country of origin (Uganda, Vietnam, and the Philippines), we observed consistently high performance across all three countries, with AUCs of 0.943 for Uganda, 0.952 for Vietnam, and 1.000 for the Philippines (Figure 5B and Table S11), confirming cross-geographical robustness. In the combined testing and validation cohorts, there were 11 patients with HIV-positive PTB and 9 HIV-positive non-PTB individuals. The model achieved 100% sensitivity (95% CI: 0.741–1.000) and 66.7% specificity (95% CI: 0.354–0.879) for PTB identification (Figure 5C), representing an encouraging improvement over the original study (90.9% sensitivity [95% CI: 0.623–0.984] and 33.3% specificity [95% CI: 0.121–0.646]). However, the wide confidence intervals reflect the limited sample size (*n* = 20), suggesting that these performance metrics may be unstable.

Biological validation showed strong positive correlation between model scores and Xpert Ultra semiquantitative grading (Figure 5D: *r* = 0.65, *p* = 7.5e-18), along with significant associations with three chest X-ray scoring systems: CAD4TB (*r* = 0.39, *p* = 6.8e-07), qXR (*r* = 0.31, *p* = 1.2e-04), and Lunit (*r* = 0.43, *p* = 4.5e-08) (Figures 5E–5G).^32^^,^^33^^,^^34^ These findings suggested that the model not only captures molecular signatures of infection but may also reflect radiological manifestations. To further evaluate the diagnostic consistency in a clinical binary framework, we conducted a Cohen’s Kappa analysis. We firstly determined the study-specific optimal cut-offs for CAD4TB (48.94), qXR (0.336), and Lunit (7.93) based on the maximum Youden Index within our cohort. Using these optimized cut-offs alongside the 5-gene pair model’s threshold of 0.5, we observed substantial diagnostic agreement, with Kappa scores of 0.56, 0.42, and 0.55 for CAD4TB, qXR, and Lunit, respectively (Figures 5H–5J). These results indicate that the host-derived molecular signature aligns well with radiological manifestations at established clinical decision points, reinforcing its reliability as a diagnostic tool. Notably, in cases where radiological and molecular predictions diverged, our 5-gene pair model provided high discriminative power in distinguishing PTB from non-PTB, suggesting that host transcriptome-based signatures can reliably capture immunological perturbations that may be subtle or ambiguous in radiological assessments. In summary, our 5-gene pair-based diagnostic model consistently demonstrates robust, generalizable performance across countries, HIV infection statuses, and various diagnostic reference standards, supporting its potential for widespread clinical implementation.

## Discussion

PTB remains a major global infectious disease with significant challenges in early and accurate diagnosis. In this study, we systematically evaluated multiple host response features at the cfRNA level and developed five diagnostic models for PTB based on distinct molecular signatures, including immune cell infiltration profiles, global transcriptional perturbation scores (expression value-/rank-based), key DEGs, and optimized 5 gene pairs. Our study provides a multidimensional framework for PTB detection and identifies the optimized 5-gene pair model as the most effective modality. In the construction of the 5-gene pair diagnostic model, a default classification threshold of 0.5 was adopted. This choice was primarily driven by the clinical priority of maximizing sensitivity, achieving 100%, to ensure early case identification and minimize missed diagnoses in the transmission chain. However, we recognize that the classification cutoff can be dynamically adjusted in different screening scenarios to optimize the trade-off between sensitivity and specificity based on local prevalence and diagnostic priorities.

Immune landscape analysis revealed profound immune remodeling in PTB, characterized by the significant depletion of adaptive immune components (naive B cells and CD8^+^ T cells, *p* < 0.05) alongside innate immune activation (neutrophils and mast cells). Kamolratanakul et al.^35^ documented sustained reduction of naive and memory B cells in active PTB, while Rozot et al.^36^ observed impaired MTB-specific CD8^+^ T cell proliferation in PTB. Neutrophils, the most abundant innate immune cells, play a dual role in PTB immunity. On one hand, they contribute to pathogen clearance via phagocytosis, reactive oxygen species production, and antimicrobial peptides release; on the other hand, their sustained activation in active PTB may exacerbate pulmonary tissue damage and immunopathology.^37^^,^^38^ Neutrophil-associated transcriptional signatures correlate with disease severity and hold potential for predicting treatment outcomes.^28^ Nevertheless, our results showed that the immune cell-based predictive model had moderate performance in cross-cohort validation (AUC 0.794–0.808), possibly due to the non-specificity of immune cell dynamics, which may reflect systemic secondary effects rather than PTB-specific signatures. These dynamics are also susceptible to age, co-infections (e.g., HIV), and host immune status, limiting their diagnostic power in complex clinical settings. Thus, while immunological features provide interpretable biological information, their translational potential relies on incorporation with more specific molecular markers.

Dual-layer cfRNA perturbation analysis further distinguished global from local feature-based approaches. From a macro perspective, the conventional molecular degree of perturbation (MDP) framework showed strong batch effects and limited specificity (validation AUC = 0.496), whereas the rank-optimized perturbation score enhanced robustness and PTB specificity via gene-ranking features (validation AUC = 0.760), suggesting that gene-pair perturbation units can better capture complex infection states. Local feature models outperformed global methods, with the 4-gene SVM classifier reaching validation AUC = 0.813, yet remained vulnerable to technical noise in absolute expression values. The gene pair paradigm decisively overcame this limitation by leveraging relative expression order, eliminating platform-specific biases, and enabling the Naive Bayes classifier built on five key gene pairs to achieve unprecedented accuracy and generalizability (validation AUC = 0.947).

Three key attributes establish gene pairs as superior PTB diagnostic biomarkers. First, the diagnostic excellence: compared with the 6-gene model proposed by Chang et al.,^17^ our gene-pair model improved all 8 performance metrics, particularly specificity, notably improving specificity by 8.0% (0.840 vs. 0.760), thereby reducing false positives and enhancing classification accuracy. Second, the technical robustness: constructing features based on relative expression order effectively bypasses quantification variability and demonstrates robustness across international and heterogeneous cohorts. The clinical relevance: gene-pair scores correlated strongly with Xpert Ultra grading (*r* = 0.65) and three chest X-ray scoring (*r* = 0.39–0.43), and achieved 100% sensitivity (95% CI: 0.741–1.000) in HIV co-infected individuals. The 5 gene pairs involve six genes, with guanylate-binding protein 5 (*GBP5*) present in all five pairs. *GBP5* was consistently identified as the most significantly upregulated DEG across both differential analysis methods and was the only shared gene between our signature and the 6-gene panel proposed by Chang et al. Multiple studies have independently confirmed the diagnostic relevance of *GBP5* in PTB.^39^^,^^40^^,^^41^ The centrality of *GBP5* in our 5-gene pair signature highlights its role as a dominant molecular anchor for PTB diagnosis. While the model utilizes *GBP5* in each pair, its robustness is derived from the collective stability of relative expression ordering against diverse partner genes. Given that *GBP5* is one of the most abundantly and consistently upregulated transcripts in PTB cfRNA across multiple geographic regions, the risk of technical non-detection is minimal. This high-abundance anchor strategy is intentionally chosen to ensure the signature remains detectable even in samples with lower RNA yields, providing a stable foundation for clinical translation in diverse settings.

The remaining five partner genes (*SERINC1, SAMD9, RHOB, UTP3,* and *ZFAND5*) of *GBP5* did not show statistically significant differential expression in PTB when analyzed individually. However, they serve as stable internal anchors, and their pairing with *GBP5* amplifies subtle expression differences into a robust discriminative signal, mitigating the inherent technical variability in cfRNA measurements. These partner genes also reduce reliance on conventional housekeeping genes and maintain robustness across different sequencing platforms and sample types, providing a reliable strategy to minimize batch effects in diverse clinical settings. Importantly, this gene-pair framework captures PTB-specific molecular characteristics. Although *GBP5* is a classic interferon-stimulated gene, it can be upregulated in various inflammatory and infectious contexts. By pairing *GBP5* with genes involved in distinct cellular processes, including vesicle trafficking, signal regulation, and RNA metabolism, our model defines a context-specific molecular fingerprint that reflects PTB-induced host immune activation and cellular remodeling.

Although the gene-pair signature was identified using cfRNA sequencing data, RNA-seq itself is not currently suitable for point-of-care testing due to cost, turnaround time, and infrastructure requirements. Importantly, the gene-pair framework is platform-agnostic and depends on relative expression ordering rather than absolute transcript abundance. This property makes it amenable to translation into targeted molecular assays, such as RT-qPCR or other amplification-based platforms, where relative Ct values can be used to infer gene-pair relationships without the requirement for universal internal reference genes. This characteristic mitigates the technical variability associated with RNA extraction efficiency in resource-limited settings. Furthermore, integrating this signature into automated molecular diagnostic systems, such as the GeneXpert framework, presents a viable pathway to reduce turnaround times and eliminate the reliance on specialized bioinformatics. Beyond established platforms, emerging technologies, including isothermal amplification and CRISPR-based diagnostics,^42^^,^^43^ could offer even more cost-effective and rapid alternatives for assessing these relative gene-pair relationships in field-based clinical settings.

In conclusion, our study systematically compared diverse cfRNA-based features for PTB diagnosis and found that PTB induces not only broad immunological remodeling but also distinct cfRNA-level perturbations. The gene-pair modeling strategy excelled in robustness, dynamic response, and resilience to HIV-related interference, ultimately identifying 5 interpretable and diagnostically valuable gene pairs. Future efforts should prioritize the rapid deployment of this stable marker panel to bridge diagnostic gaps in marginalized populations. Our findings provide a novel framework for cfRNA application in infectious disease diagnostics and lay the methodological foundation for developing more precise tools for PTB detection.

### Limitations of the study

Several limitations should be acknowledged. First, although the three cohorts analyzed in this work were separated geographically and were strictly partitioned into training, testing, and validation sets, they were derived from a single parent study. Validation in a completely independent cfRNA tuberculosis cohort generated under an unrelated study design would be essential to further confirm the generalizability of the proposed gene-pair signature. Second, the HIV-co-infected subgroup was small, yielding a wide 95% CI for both sensitivity and specificity. Consequently, the precision of this estimate is limited, highlighting the need for future validation in larger HIV-coinfected cohorts. Furthermore, while our model demonstrates robust performance in symptomatic PTB cases, its efficacy in identifying asymptomatic or subclinical PTB, which often presents with lower bacterial loads and distinct host responses, remains to be determined. Asymptomatic individuals represent a critical group in the PTB transmission chain, and evaluating our signature in this population would be an important direction for future research.

## Resource availability

### Lead contact

Requests for further information and resources should be directed to and will be fulfilled by the lead contact, Jian Huang (hj@uestc.edu.cn).

### Materials availability

This study did not generate new unique reagents.

### Data and code availability

• This article analyzes existing, publicly available data, accessible at Gene Expression Omnibus (GEO, http://www.ncbi.nlm.nih.gov/geo): GSE255071, GSE255073, GSE255074.
• All original code has been deposited at Zenodo and is publicly available at https://doi.org/10.5281/zenodo.18425286 as of the date of publication.
• Any additional information required to reanalyze the data reported in this article is available from the lead contact upon request.

## Acknowledgments

This work was supported by a grant from the 10.13039/501100001809National Natural Science Foundation of China (62501110, 62371112), Medico-Engineering Cooperation Funds from 10.13039/501100005408University of Electronic Science and Technology of China (ZYGX2022YGRH004), and the Incubation Program for Innovative Science and Technology in 10.13039/501100005408UESTC (Y03023206100209).

## Author contributions

Conceptualization, C.W.; methodology, C.W. and H.Q.; investigation, X.P. and H.L.; writing – original draft, C.W. and X.X.; writing – review and editing, M.D.; funding acquisition, M.D. and J.H.; resources, H.L. and J.H.; supervision, M.D., H.L., and J.H.

## Declaration of interests

The authors declare no competing interests.

## STAR★Methods

### Key resources table

**Table:** 

REAGENT or RESOURCE	SOURCE	IDENTIFIER
**Deposited data**		

RNA-seq data	Chang et al.	GEO: GSE255071, GSE255073, GSE255074.

**Software and algorithms**		

CIBERSORT	Newman et al.	https://doi.org/10.1038/nmeth.3337
MDP	Gonçalves et al.	https://doi.org/10.3389/fgene.2019.00971
DPS-Tool	Wu et al.	https://i.uestc.edu.cn/DPS-Tool/
cfRNA-PTB-Model	This paper	https://doi.org/10.5281/zenodo.18425286

### Experimental model and study participant details

We utilized multinational PTB cohort data from Chang et al.,^17^ strictly adhering to the original study’s data partitioning scheme to create training, testing, and validation sets. The original study by Chang et al. was approved by Institutional Review Boards at each participating site: Cornell University (IRB0145569, 1902008555); UCSF (IRB 20–32670); University of Heidelberg Ethics Committee of the Medical Faculty (S539/2020); Makerere University College of Health Sciences School of Medicine Research Ethics Committee (2017-020); Vietnam National Lung Hospital Ethical Committee for Biological Medical Research (566/2020/NCKH); and De La Salle Health Sciences Institute Independent Ethics Committee (2020-33-02-A).

### Method details

#### Data preprocessing

Raw FASTQ files underwent quality assessment using FastQC (v0.11.9) and MultiQC (v1.13), followed by adapter removal and low-quality reads filtering with Fastp (v0.22.0). Clean reads were aligned to the GRCh38 human reference genome using STAR (v2.7.10b), with gene expression quantification performed by HTSeq (v2.0.2). Transcripts Per Million (TPM) normalized expression values were annotated to Gene Symbols via Ensembl GTF files. For genes with multiple Ensembl IDs, the entries showing highest mean expression across samples were retained. Final analysis included 21,637 genes expressed in ≥20% of samples and shared across all datasets.

#### CIBERSORT immune cell profiling

Immune cell composition was deconvoluted using CIBERSORT^44^ (R package v0.1.0) with LM22 signature matrix to estimate the relative abundance of 22 immune cell subtypes in the training samples. TPM-normalized RNA-seq data underwent 1000 permutations with quantile normalization disabled to accommodate sequencing data characteristics. The relative proportions of 22 immune cell subtypes summed to 1 in each sample, and subsequent intergroup comparisons and correlation analyses were performed based on these results.

#### Molecular perturbation analysis using expression value

To quantify the global gene expression deviation of PTB samples versus non-PTB controls in the training cohort, the MDP algorithm developed by Gonçalves et al. was applied.^25^ First, reference distributions (*μ* ± *σ*) were established for each gene using non-PTB samples. For each PTB sample, the gene-level MDP (gMDP) was calculated as (sample value - *μ*)/*σ*. Subsequently, genes with |gMDP| < 1 were filtered out to eliminate random noise, and the top 1% of genes showing the maximal differential gMDP between PTB and non-PTB groups were selected. Finally, the sample-level MDP (sMDP) was derived by averaging the absolute gMDP values of the selected genes. All analyses were performed using the R mdp package (v1.26.0).

#### Molecular perturbation analysis using expression rankings

Building upon the MDP framework, we developed the Disease Perturbation Scoring Tool (DPS-Tool) to evaluate the global transcriptional perturbations of PTB versus non-PTB samples in the training set, based on gene expression rank alterations.^45^ The core logic involves identifying reversed gene pairs between groups.^46^^,^^47^ The extraction of discriminative gene pairs followed a systematic five-step procedure. First, all genes were combined pairwise to form an exhaustive library of candidate gene pairs. For each pair (comprising Gene i and Gene j), the frequency of the relative expression order (Gene i < Gene j) was calculated independently within the PTB and non-PTB groups. The disparity score for each gene pair was then derived by computing the absolute difference in these frequencies between the two groups. Subsequently, gene pairs were filtered based on application-specific thresholds to balance sensitivity and specificity. Specifically, a threshold of 0.30 was applied during rank-based perturbation analysis to capture a broader range of candidate pairs for global transcriptional disruption assessment. In contrast, a more stringent threshold of 0.65 was utilized for constructing the final PTB diagnostic model to prioritize pairs with high biological relevance and robust discriminative power. Finally, redundancy filtration was performed according to the analysis requirements: for rank-based perturbation analysis, if a single gene appeared in multiple pairs, only the pair with the highest disparity score was retained to minimize feature redundancy; however, for the diagnostic signature, all gene pairs exceeding the threshold were preserved without de-duplication to capture the multi-dimensional diagnostic signals of the host response.

After obtaining the signature gene pairs, the perturbation score (*DPS-Tool Score*) for an individual sample *j* is derived by quantifying the cumulative rank disruptions:

(Equation 1) $$\begin{array}{c} {DPS-Tool\;Score}_{j}=S_{j} \end{array}$$

Where *S*_*j*_ represents the number of those gene pairs that exhibit a reversed state in sample *j*. For instance, if a signature consists of three gene pairs (*G1-G2, G2-G3, and G1-G3*) and a sample shows reversals in only two of them (e.g., *G1* > *G2* and *G1* > *G3* observed), the *DPS-Tool Score* for that sample would be 2.

#### Differential expression and functional enrichment analysis

To identify DEGs, we employed two complementary approaches: the linear model-based limma method^48^ (R package limma v3.54.0) and the non-parametric Wilcoxon rank-sum test. A dual-filtering strategy was adopted to improve robustness. To further investigate the biological processes and pathways involved, enrichment analyses were performed based on Gene Ontology (GO), the KEGG, and the Reactome pathway database using the R packages clusterProfiler (v4.15.0.3)^49^ and ReactomePA (v1.42.0).^50^ These annotations provided a comprehensive view of DEGs enrichment in biological processes, molecular functions, cellular components, and signaling pathways, helping to elucidate potential disease mechanisms and key regulatory networks.

#### Construction of multimodal feature-based PTB diagnostic model

We established five PTB diagnostic models using distinct feature modalities. For immune cell profiles, global transcriptional perturbation features, and rank-based global perturbation features, logistic regression (LR)-based models with default parameters were constructed.

In local key DEGs-based feature modeling, the process followed a systematic pipeline. First, in model selection, the feature matrix containing all 45 DEGs was first input into 10 commonly used machine learning algorithms, including SVM, LR, Extreme Gradient Boosting (XGBoost), Random Forest (RF), k-Nearest Neighbors (KNN), Naive Bayes, Soft Voting, Stacking, Multi-Layer Perceptron (MLP), and Convolutional Neural Network (CNN), to systematically evaluate and select the algorithm most appropriate for the underlying data characteristics. To ensure a fair comparison, all classifiers were trained using default parameters, and performance was evaluated via 5-fold CV on the training set. The best-performing algorithm was selected based on average validation performance. Subsequently, feature importance scores and corresponding ranks for each DEG were calculated using four widely adopted methods: Maximal Information Coefficient (MIC),^51^ GBDT,^52^ Relief,^53^ and adjusted *p*-values. Third, to optimize the feature subset, IFS^54^^,^^55^ was applied to iteratively add features to the model one by one in descending order of their importance rank. The optimal feature subset was identified at the inflection point where the 5-fold CV performance on the training set reached its peak, effectively balancing diagnostic sensitivity and model parsimony. Final predictive models were built using the selected algorithm and optimal feature set, followed by model optimization through randomized hyperparameter search. All workflows were implemented in Python 3.10.13.

Gene pair-based feature modeling followed similar procedures as DEG-based methods, including model selection, feature selection, hyperparameter tuning, and final model building, with critical differences in feature processing: Raw expression matrices were converted to binary relational matrices (columns labeled ‘*Gene*_*i*_|*Gene*_*j*_*’*, encoded as 1 for *Gene*_*i*_ < *Gene*_*j*_, 0 for equality, and −1 for *Gene*_*i*_ > *Gene*_*j*_). Additionally, feature selection incorporated an additional gene pair-specific score (inter-group frequency difference of *Gene*_*i*_ < *Gene*_*j*_) alongside MIC, GBDT, and Relief methods to prioritize pairs with the most stable and significant discriminative power.

#### Model performance evaluation

Several evaluation metrics were employed to comprehensively assess model performance, including accuracy (ACC), sensitivity (SN/recall), specificity (SP), precision, F1 score, and Matthews Correlation Coefficient (MCC).

(Equation 2) $$\begin{array}{c} \left\{ \begin{array}{c} ACC=\frac{TP+TN}{TP+TN+FP+FN}\\ SN\;\left(recall\right)=\frac{TP}{TP+FN}\\ SP=\frac{TN}{TN+FP}\\ precision=\frac{TP}{TP+FP}\\ F1\;score=2\times \frac{precision\times recall}{precision+recall}\\ MCC=\frac{TP\times TN-FP\times FN}{\sqrt{\left(TP+FN\right)\times \left(TN+FN\right)\times \left(TP+FP\right)\times \left(TN+FP\right)}} \end{array}\right. \end{array}$$

where TP, FP, TN, and FN denote the number of true positives, false positives, true negatives and false negatives, respectively. Additionally, the area under the receiver operating characteristic curve (AUC) was also used to evaluate the predictive performance of the model.

### Quantification and statistical analysis

For intergroup comparisons, differences in age distribution, relative abundance of immune cell types, global transcriptional perturbation features, and rank-based global perturbation features were assessed using the Wilcoxon rank-sum test, whereas differences in sex distribution were evaluated with the chi-square test. A *p*-value <0.05 was considered statistically significant.

For correlation analyses, the relationships between the relative abundance of immune cells, global transcriptional perturbation features, rank-based global perturbation features, and the gene-pair score (i.e., the model-predicted probability) with the semi-quantitative levels of MTB reported by the Xpert MTB/RIF Ultra assay were assessed using the Kendall rank correlation test. Correlations between the gene-pair score and three chest imaging scores were also examined using the same method. A significance threshold of *p* < 0.05 was applied, and all statistical analyses were conducted in R version 4.2.2.

## Supplemental information

Table S1. Baseline characteristics and clinical demographics of participants across the training, testing, and external validation cohorts

Table S2. The results of differential expression analysis using limma

Table S3. The results of differential expression analysis using the Wilcoxon rank-sum test

Table S4. The intersection of differentially expressed genes between the two methods

Table S5. The importance ranking of pulmonary tuberculosis-related genes based on four methods

Table S6. Gene-pair threshold selection and comparison of feature numbersur methods

Table S7. Reverse gene pairs

Table S8. The importance ranking of reverse gene pairs based on four methods

Table S9. Functional annotation of the 6 unique genes in the 5-gene pair model

Table S10. Performance and confusion matrices of the 5-gene pair model across the independent validation

Table S11. Performance and confusion matrices of the 5-gene pair model across countries
